# Construction and effect evaluation of a prediction model for malnutrition risk in patients recovering from stroke

**DOI:** 10.3389/fnut.2026.1776272

**Published:** 2026-06-22

**Authors:** Li-Ya Gong, Yan Wang, Jian Shi, Xiao-Dong Nie, Yue-Lin Ma

**Affiliations:** Clinical Nutrition Department, The Second Affiliated Hospital of Hebei Medical University, Shijiazhuang, Hebei, China

**Keywords:** malnutrition, nomogram, recovery period, risk prediction model, stroke

## Abstract

**Background:**

The incidence of malnutrition in stroke patients during recovery is high, which seriously affects the rehabilitation outcome. Existing general nutrition screening tools have limited predictive power for this specific population.

**Purpose:**

To construct and validate a prediction model specifically for assessing the risk of malnutrition in patients with stroke during recovery.

**Method:**

A total of 262 patients with stroke in recovery stage were retrospectively enrolled and divided into training set (*n* = 176) and validation set (*n* = 86) according to admission time. Nutritional risk was defined as nutritional risk screening 2002 (NRS-2002) score ≥3. Clinical and laboratory indicators were collected, and Logistic regression analysis was used to screen independent predictors and construct a nomogram model. The discrimination and calibration of the model were evaluated by receiver operating characteristic (ROC) curve and calibration curve, and Bootstrap internal verification and independent temporal validation were performed.

**Results:**

Multivariate analysis identified age, modified Barthel index (MBI) score and albumin level as independent predictors of malnutrition. The AUC of the nomogram model was 0.869 (95%CI: 0.817–0.922) in the training cohort and 0.878 (95%CI: 0.808–0.949) in the validation cohort. The calibration curve showed good agreement between the predicted risk and the actual risk.

**Conclusion:**

This study successfully constructed and verified a risk prediction model for malnutrition in stroke recovery patients including age, functional status and albumin level. As a complementary tool to the existing screening method NRS-2002, the model has good predictive power and clinical applicability. It facilitates early identification of high-risk patients and provides an evidence-based approach to individualized nutritional intervention.

## 1 Introduction

Stroke is one of the main causes of disability and death worldwide, and its high incidence, high disability rate and high recurrence rate have become a serious public health problem (1–3). In China, stroke ranks first in the cause of adult death and disability, bringing heavy disease and economic burden to patients, families and society (4–6). With the improvement of medical standards, more stroke patients have entered the recovery stage, which is not only a critical period for neurological reconstruction and rehabilitation, but also an important stage for the persistence of various complications risk (7, 8). Among them, malnutrition, as a common and serious complication after stroke, is particularly prominent in patients during the recovery period. Studies have shown that up to 62% of stroke patients develop malnutrition throughout the course of their illness (9). During the recovery period, the risk of malnutrition is further aggravated due to multiple factors such as persistent dysphagia, neurological deficits, decreased mobility, depression, and drug side effects (10–12). Malnutrition not only directly impairs the patient’s immune function, delays tissue repair, and reduces muscle strength and functional status, but also is significantly associated with increased risk of infection, prolonged hospital stay, poor rehabilitation, increased readmission rate, and increased mortality (13–15). Therefore, early and accurate identification of patients with high risk of malnutrition and implementation of targeted interventions are of vital significance for improving the prognosis of patients, improving the quality of life, and reducing the medical burden of society.

At present, clinical screening of nutritional risk in stroke patients mostly relies on universal assessment tools, such as Nutritional Risk Screening 2002 (NRS 2002), Malnutrition Universal Screening Tool (MUST), Mini-nutritional Assessment short Form (MNA-SF) (16, 17). These tools are generalizable across inpatient populations and have shown some predictive value in multiple studies (18, 19). However, it is not designed for the pathophysiological characteristics of patients in the recovery stage of stroke, which may lead to insufficient sensitivity and specificity, or unable to accurately quantify the risk level, thus affecting the timeliness and accuracy of the intervention. In recent years, with the development of precision medicine and prediction model methodology, the construction of disease risk prediction model has become a hot spot in clinical research (20, 21). By integrating multi-dimensional clinical indicators and using statistical methods to establish a prediction model, it can quantify the risk probability of individual patients, realize risk stratification, and provide an intuitive and quantitative tool for clinical decision-making. Among the various forms of presentation, nomogram has significant advantages because it transforms complex regression equations into an intuitive, easy-to-use interface, enables continuous risk assessment rather than binary classification, and facilitates shared decision making. However, there is a lack of specially constructed and rigorously validated malnutrition risk prediction models for this specific population in the recovery stage of stroke. Therefore, this study aims to systematically analyze the related influencing factors of malnutrition risk in patients with stroke in recovery period, screen the independent predictors, construct a prediction model for malnutrition risk in patients with stroke in recovery period, and comprehensively evaluate its predictive efficacy. It is hoped that the results of this study will help clinical medical staff to identify high-risk patients early and accurately, achieve preventive management of malnutrition, and provide evidence-based basis for the formulation of individualized nutritional support and rehabilitation management strategies for stroke patients in the recovery period.

## 2 Materials and methods

### 2.1 Patient

A total of 262 patients with stroke in recovery stage admitted to the Department of Rehabilitation Medicine of the Second Affiliated Hospital of Hebei Medical University from January 2022 to December 2024 were retrospectively collected. According to the time of admission, they were divided into two groups: training set (176 patients from January 2022 to December 2023) and validation set (86 patients from January 2024 to December 2024). Nutritional risk was defined as NRS-2002 score ≥3, and no nutritional risk was defined as NRS-2002 score < 3. NRS-2002 was selected as the reference standard for nutritional risk screening because it is widely used in hospitalized patients in China, recommended by major clinical nutrition guidelines such as ESPEN and CSPEN, and has been validated in the stroke population. It served as the basis for identifying high-risk patients in this study and was compared with newly developed models in terms of discrimination and calibration, not as a “gold standard” in pathophysiology, but as a clinically feasible screening benchmark. The study was approved by the ethics committee of our hospital.

### 2.2 Inclusion and exclusion criteria

Inclusion criteria: (1) patients met the diagnostic criteria of stroke and were diagnosed with stroke; (2) convalescent inpatients whose course of disease was within 2 weeks to half a year after onset; (3) clear consciousness; (4) age ≥18 years old. Exclusion criteria: (1) repeat admission; (2) patients with severe heart, liver, kidney and other organ failure; (3) patients with tuberculosis, malignant tumor and psychogenic anorexia; (4) end of life patients; (5) those with missing key data.

### 2.3 Data collection

A retrospective study was conducted, and data were collected by reviewing the electronic medical records of patients. (1) General information: age, gender, body mass index, type of residence, type of stroke, history of hypertension, history of diabetes, history of smoking, and history of drinking. (2) Disease-related data: NIHSS score, modified Barthel index (MBI), and nutritional support methods. The NIHSS score was used to assess the degree of neurological deficit, with a total score of 42, and the higher the score, the more severe the neurological deficit (22). The MBI was used to assess the ability of daily living of patients. The total score ranged from 0 to 100, and the lower the score, the worse the ability of daily living (23). All these scores were assessed by physicians on admission and entered into the electronic medical record system. (3) Laboratory data: white blood cell count, red blood cell count, albumin level. Laboratory data were measured for the first time within 24 h after admission. Patients with missing key data were excluded from the analysis as specified by the exclusion criteria. Key data were defined as the basic variables required for outcome assessment and core predictor variables. This approach ensured the integrity of the analyses for all enrolled patients.

### 2.4 Statistical analysis

The sample size was not precalculated on the basis of a power analysis but was determined on the basis of the total number of patients who were consecutively admitted to a single center during a given period and met the inclusion and exclusion criteria. The number of malnutrition events in the training set was 112, but only 3 predictive variables were included in the final model, and the event-variable ratio (EPV) was much greater than 10, which met the sample size requirement. SPSS 25.0 software was used to analyze the obtained data. Measurement data were expressed as mean ± standard deviation and compared by *t* test. Count data were expressed as cases or percentages and compared using the chi-square test or Fisher’s exact test. Logistic regression analysis identified independent factors influencing the occurrence of malnutrition. Multicollinearity between all candidate predictors was assessed with the use of variance inflation factor (VIF) analysis. R4.2.1 software was used to construct the nomogram model, and the receiver operating characteristic (ROC) curve was used to evaluate the discrimination of the model. The Bootstrap method (self-sampling number was 2000) was used for internal verification. The calibration curve was used to evaluate the calibration of the model. In addition, based on the constructed nomogram model, ROC analysis was performed on the data of the validation set to conduct temporal validation of the model. The optimal cut-off value for classifying high-risk malnutrition was determined using the Youden index from the ROC curve of the training set. Continuous predictors were transformed into clinically meaningful categorical variables and integer scores were assigned proportionally to the regression coefficients according to tertiles or clinical thresholds. *p* < 0.05 was considered statistically significant.

## 3 Results

### 3.1 Baseline data

A total of 262 patients were included in this study. There were 176 cases in the modeling group, including 110 males and 66 females, with an average age of (63.86 ± 12.76) years, and 112 cases (63.64%) were at risk of malnutrition. In the validation group, there were 48 males and 38 females, with an average age of (64.51 ± 11.79) years, and 47 (54.65%) of them were at risk of malnutrition. By comparison of baseline data, there was no significant difference in general data between the modeling group and the validation group (*p* > 0.05), and the homogeneity was good, and the model could be verified in the validation group (Table 1).

**Table 1:** Baseline characteristics of patients recovering from stroke.

Characteristic	Modeling group (*N* = 176)	Verification group (*N* = 86)	*p* value
Gender, *n* (%)			0.299
Male	110 (62.50)	48 (55.81)	
Female	66 (37.50)	38 (44.19)	
Age, years	63.86 ± 12.76	64.51 ± 11.79	0.814
Stroke types, *n* (%)			0.497
Cerebral infarction	114 (64.77)	52 (60.47)	
Cerebral hemorrhage	62 (35.23)	34 (39.53)	
History of diabetes, *n* (%)	58 (32.95)	26 (30.23)	0.658
History of hypertension, *n* (%)	120 (68.18)	63 (73.26)	0.401
Smoking history, *n* (%)	76 (43.18)	40 (46.51)	0.610
Drinking history, *n* (%)	80 (45.45)	43 (50.00)	0.489
Housing type, *n* (%)			0.872
Living alone	18 (10.23)	8 (9.30)	
With spouse	82 (46.59)	43 (50.00)	
With parents/children	76 (43.18)	35 (40.70)	
Risk of malnutrition, *n* (%)			0.162
Yes	112 (63.64)	47 (54.65)	
No	64 (36.36)	39 (45.35)	

### 3.2 Univariate analysis of malnutrition risk in the modeling group

Univariate analysis of the data of the modeling group showed that age, nutritional support method, NIHSS score, MBI score, hemoglobin count, and albumin level were the influencing factors of malnutrition risk in patients with stroke in the recovery period (*p* < 0.05). Age, MBI score and albumin level were significant influencing factors (*p* < 0.001) (Table 2).

**Table 2:** Univariate analysis of risk factors for malnutrition during stroke recovery period in modeling group.

Characteristic	Malnutrition risk group (*N* = 112)	Non-malnutrition risk group (*N* = 64)	*χ*^2^/*t*	*p* value
Gender, *n* (%)			0.733	0.392
Male	68 (60.71)	43 (67.19)		
Female	44 (39.29)	21 (32.81)		
Age, years	63.20 ± 11.23	52.42 ± 9.60	6.824	<0.001
BMI, kg/m^2^			3.983	0.137
<18.5	12 (10.71)	3 (4.69)		
18.5–24.9	72 (64.29)	50 (78.13)		
≥25.0	28 (25.00)	11 (17.18)		
Housing type, *n* (%)			5.694	0.058
Living alone	11 (9.82)	5 (7.81)		
With spouse	57 (50.89)	22 (34.38)		
With parents/children	44 (39.29)	37 (57.81)		
Smoking history, *n* (%)	47 (41.96)	29 (45.31)	0.186	0.666
Drinking history, *n* (%)	46 (41.07)	34 (53.13)	2.387	0.122
History of diabetes, *n* (%)	34 (30.36)	24 (37.50)	0.941	0.332
History of hypertension, *n* (%)	72 (64.29)	48 (75.00)	2.156	0.142
Nutritional support methods, *n* (%)			5.023	0.025
Oral feeding	95 (83.93)	60 (95.31)		
Tube feeding	17 (16.07)	4 (4.69)		
NIHSS score, points	8.07 ± 3.92	5.91 ± 9.13	2.285	0.030
MBI score, points			18.04	< 0.001
<40	33 (29.47)	34 (53.13)		
40–60	29 (25.89)	21 (32.81)		
>60	50 (44.64)	9 (14.06)		
White blood cells (10^9^/L)	6.68 ± 2.02	6.25 ± 1.88	0.694	0.492
Hemoglobin (g/L)	122.10 ± 11.36	134.90 ± 14.52	3.245	0.002
Albumin (g/L)	36.87 ± 2.91	39.32 ± 2.77	5.677	< 0.001

BMI, Body Mass Index; MBI, Modified Barthel Index; NIHSS, National Institute of Health Stroke Scale.

### 3.3 Multivariate analysis of malnutrition risk in the in the modeling group

The results of the VIF analysis showed that all candidate variables had VIF values below 2.0, well below the conventional thresholds of 5 or 10. This indicates that there is no significant multicollinearity between the candidate predictors. The variables with statistically significant differences in univariate analysis were entered into the multivariate logistic regression model by stepwise forward selection method based on likelihood ratio test. The results showed that age, MBI score and Alb level were independent influencing factors of malnutrition risk in patients with stroke in recovery stage (*p* < 0.001) (Table 3). Variables including nutritional support method, NIHSS score, and hemoglobin, although significant in univariable analysis, were not retained as independent predictors in the final model.

**Table 3:** Multivariate analysis of risk of malnutrition during stroke recovery period in modeling group.

Characteristic	*β*	S.E.	Wald	*p*	OR	95% CI
Age (years)	0.058	0.018	10.441	< 0.001	1.060	1.023–1.099
MBI score, points	−0.045	0.012	14.063	< 0.001	0.956	0.934–0.979
Albumin (g/L)	−0.328	0.085	14.882	< 0.001	0.720	0.609–0.851

MBI, Modified Barthel Index; Reference categories, for continuous variables (age, MBI, albumin), ORs represent the change in odds per unit increase. OR < 1 for MBI and albumin indicates a protective effect. Only variables with *p* < 0.05 in multivariable logistic regression were retained as independent predictors in the final nomogram model. Variables that were significant in univariable analysis but not retained in the final model include nutritional support method (*p* = 0.116), NIHSS score (*p* = 0.064), and hemoglobin (*p* = 0.056).

### 3.4 Development of a risk scoring model for malnutrition

**Figure 1:**
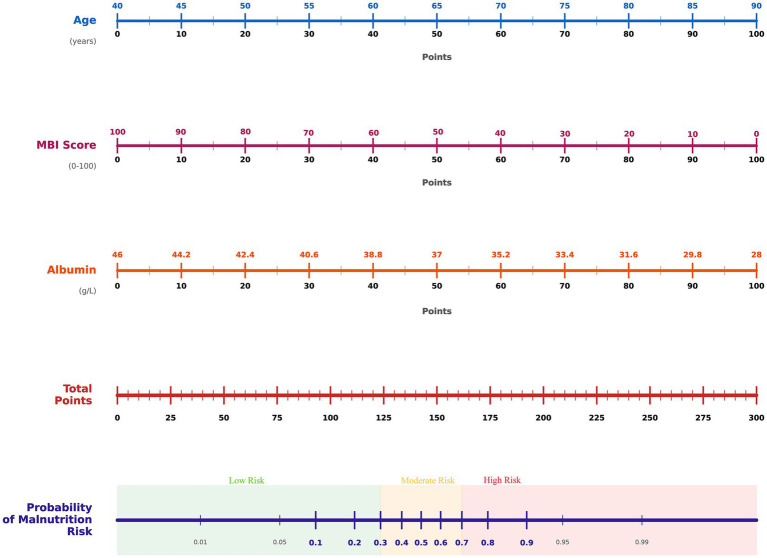
Nomogram for the prediction model of nutritional risk in convalescent stroke patients. Add the points for all three variables and find this total on the total points axis. A line was drawn down the risk axis to obtain the predicted probability of malnutrition risk. For example, for a 70-year-old patient with an MBI score of 45 and an albumin level of 37 g/L, total score = 60 + 55 + 50 = 165. This patient has a total score of more than 160, indicating high risk. It should be noted that extremely low albumin (<28 g/L) may indicate serious underlying pathology, beyond the scope of this predictive model, and requiring specialist referral.

The independent influencing factors screened by multivariate analysis were used to construct a risk nomogram model for malnutrition in convalescent stroke patients. In the nomogram, each predictor is assigned a point value based on its regression coefficient. According to the predictive factors, the corresponding scores can be found in the nomogram, and the corresponding value of the sum of each score is the probability of malnutrition (Figure 1). Low risk (total points <120 or predicted probability <0.3): standard nutritional monitoring and routine dietary guidance are sufficient; Moderate risk (total points:120–160 or predicted probability 0.3–0.7): strengthen nutritional assessment, early consultation with dietitian, and consider nutritional supplementation; High risk (total points >160 or predicted probability >0.7): immediate comprehensive nutritional intervention, close participation of dietitians, close monitoring of oral feeding and biochemical indicators, and assessment of the need for enteral nutrition support. The risk system supports two modes of use: Rapid screening: simply calculate a total score and match risk categories (low/medium/high); Detailed evaluation: For subtle clinical decisions, exact probabilities can still be read out from the nomogram.

### 3.5 Validation of a nutritional risk prediction model

**Figure 2:**
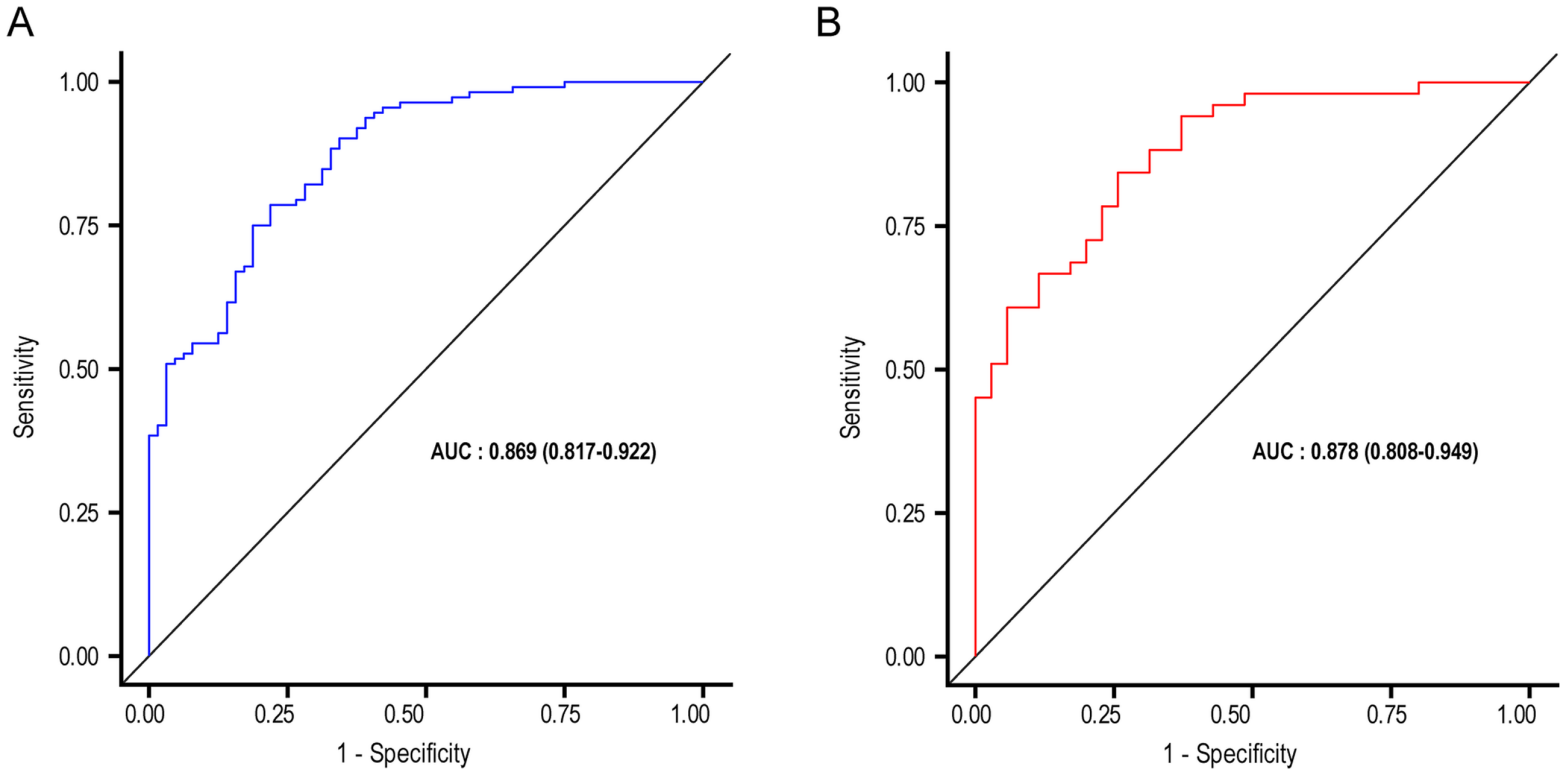
ROC curve of the prediction model. **(A)** Training set (AUC = 0.869); **(B)** Validation set (AUC = 0.878); the diagonal reference line represents the performance of a random classifier (AUC = 0.500).

**Figure 3:**
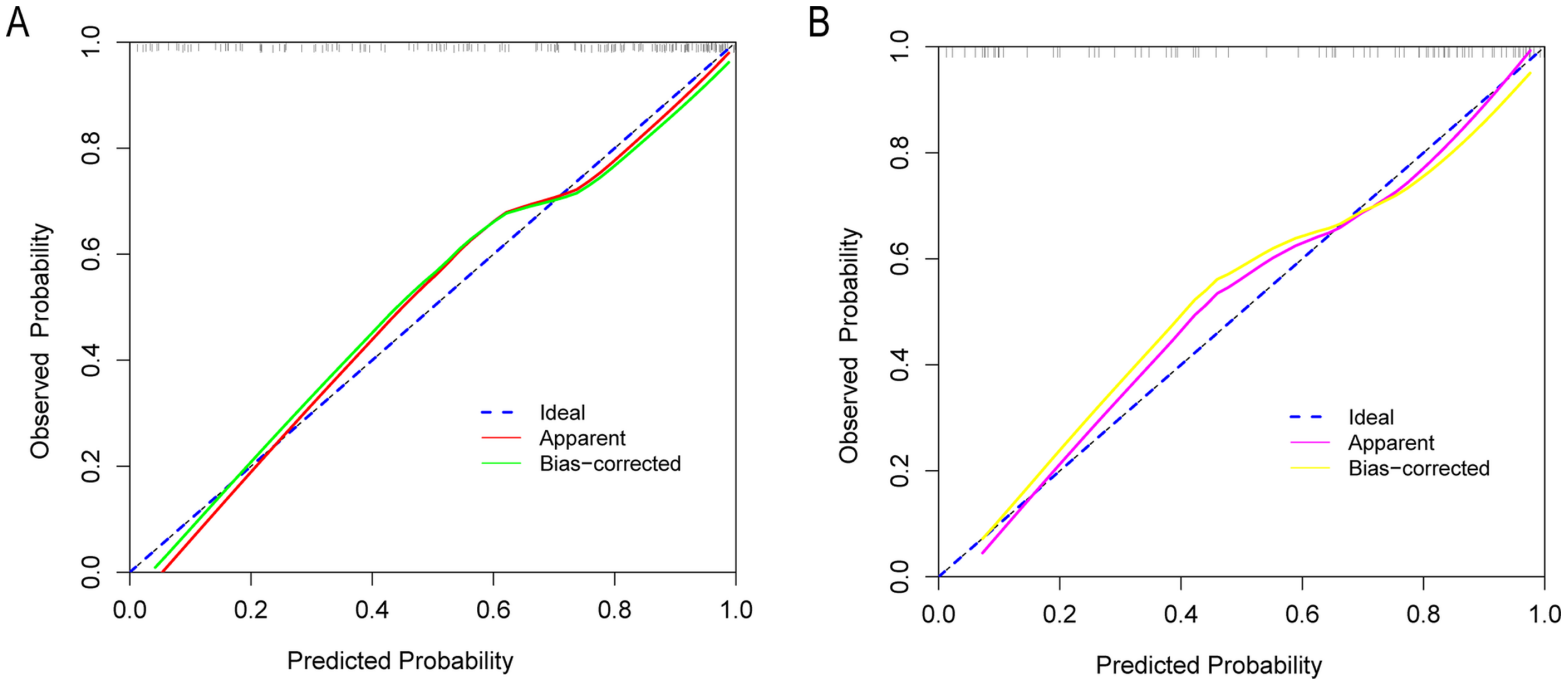
Calibration curve of the predictive model. **(A)** Training set; **(B)** Validation set; the dashed line represents perfect calibration (predicted probability = observed frequency), while the solid line with shaded confidence interval illustrates the model’s calibration performance. Good calibration indicates that the predicted probabilities accurately reflect the true likelihood of malnutrition events.

AUC was used to evaluate the discrimination of the prediction model. The ROC curve of the training set showed that the AUC was 0.869 (95%CI: 0.817–0.922), and the AUC of the validation set was 0.878 (95%CI: 0.808–0.949). The prediction model performed better than 0.8 in both the training set and the validation set, indicating that the model had good discrimination. Meanwhile, after 2000 bootstrap resamples within the training cohort, the AUC reached 0.850 (95% CI: 0.656–0.846), consistent with the original curve. This indicates that both internal and temporal validation confirm the high discriminatory power and excellent predictive performance of this prediction model (Figure 2). Based on ROC analysis, Youden index was used to determine the best cut-off value. For the training set, the best cut-off was 0.542. At this threshold, the model had a sensitivity of 82.1% (95% CI: 0.739–0.887), a specificity of 81.3% (95% CI: 0.695–0.899), and a PPV of 87.7% (95% CI: 0. 798–0.932), NPV 74.3% (95% CI: 0.630–0.833). In the validation set, the best cut-off was 0.538, the sensitivity was 85.1% (95% CI: 0.717–0.938), the specificity was 82.4% (95% CI: 0.665–0.925), and the PPV was 83.3% (95% CI: 0.698–0.925) and NPV 84.2% (95% CI: 0.692–0.934) (Table 4). The calibration curve in the training set was basically consistent with the ideal curve, and the calibration curve in the validation set was also basically consistent with the ideal curve. This indicated that the prediction model had good calibration through internal and temporal validation, and its risk of malnutrition in convalescent stroke patients was highly consistent with the actual risk (Figure 3). The overall goodness-of-fit of the final model was reported using the Hosmer–Lemeshow test. In the training set, the chi-square value of the Hosmer–Lemeshow test was 6.847 (*p* = 0.553), indicating that there was no statistically significant difference between the predicted and observed risks, suggesting that the model fit was good.

**Table 4:** Discriminative performance and optimal cutoff of the prediction model.

Dataset	Training set	Validation set
AUC (95% CI)	0.869 (0.817–0.922)	0.878 (0.808–0.949)
Sensitivity (%)	82.1 (0.739–0.887)	85.1 (0.717–0.938)
Specificity (%)	81.3 (0.695–0.899)	82.4 (0.665–0.925)
PPV (%)	87.7 (0.798–0.932)	83.3 (0.698–0.925)
NPV (%)	74.3 (0.630–0.833)	84.2 (0.692–0.934)
Accuracy (%)	81.8 (0.752–0.873)	83.7 (0.743–0.908)
Optimal cutoff	0.542	0.538
Youden index	0.634	0.672

PPV, positive predictive value; NPV, negative predictive value. Values in parentheses represent 95% confidence intervals. The optimal cutoff was determined by the maximum Youden index.

## 4 Discussion

This study successfully constructed and validated a nomogram prediction model specifically for assessing the risk of malnutrition in this group by retrospectively analyzing the clinical data of 262 patients in the recovery period of stroke. The model included three independent predictors: age, modified Barthel index score, and albumin level. The results of internal and temporal validation showed good discrimination and calibration in both the training set and the validation set, indicating that as a practical tool, it has high prediction accuracy and clinical applicability.

Multivariate logistic regression analysis identified that older age, lower MBI score and lower serum albumin level were independent risk factors for malnutrition. These findings are consistent with existing evidence on malnutrition after stroke. Advanced age is a recognized risk factor for malnutrition in a variety of chronic diseases and is often attributed to age-related physiological decline, such as decreased appetite, impaired taste and smell, and comorbidities that affect nutrient intake and absorption (24, 25). During stroke recovery, older patients often show more severe neurological deficits and slower functional recovery, which further hinders adequate oral intake and the ability to self-feed (26). The model in the present study quantifies this risk and shows that the odds of malnutrition increase by 6% for each one-year increase in age. The inverse association between the MBI score and the risk of malnutrition underscores the critical link between functional independence and nutritional status. Patients with severely limited ability to perform activities of daily living often require assistance with feeding, which may result from inadequate oral intake due to the timing of feeding, the quality of the assistance, or negative emotions of the patient (27, 28). In addition, decreased mobility leads to disuse atrophy of muscle and sarcopenia, thus creating a vicious cycle. This highlights the need to incorporate nutritional assessment into a comprehensive functional assessment during stroke rehabilitation. Albumin level, as an important indicator reflecting the nutritional status of the body, was also confirmed to be closely related to the risk of malnutrition in this study. Albumin is not only involved in the maintenance of plasma colloid osmotic pressure, but also plays an important role in immune regulation and anti-oxidation (29). Low albumin level usually indicates inadequate nutrient intake or chronic consumption, which may be related to inflammatory response, abnormal liver function and other factors (30). During stroke recovery, patients often present with post-stroke inflammatory responses and varying degrees of organ dysfunction, which may independently affect albumin concentrations. Thus, although albumin level was identified as an independent predictor of malnutrition risk in this study, it may partly reflect the combined effects of poor nutrient intake and underlying inflammatory/catabolic states. Clinicians should interpret this predictor with caution and consider albumin as a component of comprehensive nutritional assessment rather than an independent diagnostic criterion.

Related studies have confirmed the high prevalence of malnutrition after stroke and have identified similar risk factors, including age, stroke severity, dysphagia, and functional status (31, 32). The key limitation of widely used tools such as NRS 2002 and MNA-SF is their lack of specificity for the unique pathophysiology and recovery trajectory of stroke. Instead, the nomogram model in this study outputs continuous risk probabilities based on three readily available predictors: age, MBI score, and albumin level. This allows clinicians to categorize patients into risk classes and tailor monitoring and intervention accordingly. In addition, the model allows for dynamic reassessment: risk estimates can be updated in real time as a patient’s functional status (MBI) or albumin level improves or decreases. NRS-2002 or similar generic tools do not provide such functionality. Given that nutritional status and functional capacity change continuously during rehabilitation, it is recommended that the nomogram be reevaluated at 2-week intervals or when there is a significant clinical change. The model requires no additional cost or specialized equipment, and its visualization format does not require computational software, so it can be used in resource-limited Settings while supporting shared decision making among clinicians, patients, and caregivers. It is worth noting that the following factors should be paid attention to for the successful implementation of the nomogram model: training rehabilitation personnel should conduct accurate MBI assessment, because inter-rater reliability may affect the consistency of scores; Considering that pre-analytical variables may affect the levels, the albumin measurement protocols of each laboratory need to be standardized. Integrated with electronic health record systems, the nomogram can be programmed as a clinical decision support tool that automatically calculates risk probabilities and triggers alerts for high-risk patients. In the future, more validation studies are needed to evaluate the real-world effectiveness. Unfortunately, this study did not directly compare the predictive performance of the model and NRS-2002 in the same cohort. However, the available evidence showed that the AUC of NRS-2002 for predicting malnutrition in stroke patients was usually between 0.70 and 0.80, while the model in this study had an AUC of 0.869 in the training set and 0.878 in the validation set, indicating at least comparable, if not better discrimination. More importantly, the main value of the model in this study is not to replace NRS-2002 but to complement NRS-2002 by providing detailed risk quantification and stroke-specific risk factors to support precision nutrition management in the recovery period.

### 4.1 Limitation

This study also has several limitations. It employed a retrospective design with a relatively small sample size, and all data were sourced from a single medical institution, which may limit the model’s generalizability. Although we incorporated a relatively comprehensive set of clinical variables, unmeasured confounding factors such as severity of dysphagia, cognitive impairment, depression scores, inflammatory state, socioeconomic factors, and dietary intake records may also influence nutritional status. Age is a component of NRS-2002, and age was included in the model as a predictor in this study; this overlap could theoretically lead to overestimation of the discriminative performance of the model. The study did not directly compare the predictive performance of the nomogram with that of the reference tool (NRS-2002) in the same validation cohort. Therefore, while the model itself shows good discrimination and calibration, its additional clinical benefit over NRS-2002 requires further prospective comparative studies. Furthermore, the follow-up period was limited to the inpatient recovery phase, and its predictive value for long-term nutritional outcomes after discharge remains unclear. Future studies can conduct multicenter and prospective studies to further verify the stability and applicability of the model. Multi-dimensional data were integrated to optimize the model, and the long-term follow-up data were used to further evaluate the clinical value of the model.

## 5 Conclusion

This study successfully constructed and validated a nutritional risk prediction model for stroke patients in the recovery phase based on age, MBI scores, and albumin levels. As a complementary tool to existing universal screening methods (NRS-2002), this model offers excellent discrimination and calibration, enabling early clinical identification of high-risk patients and individualized risk quantification. It provides evidence-based support for personalized, preventive nutritional management, with the potential to improve patient outcomes, enhance rehabilitation effectiveness, and reduce the burden on healthcare systems.

## Data Availability

The original contributions presented in the study are included in the article/supplementary material, further inquiries can be directed to the corresponding author/s.
